# A Novel Bacterial Pathogen of *Biomphalaria glabrata*: A Potential Weapon for Schistosomiasis Control?

**DOI:** 10.1371/journal.pntd.0003489

**Published:** 2015-02-26

**Authors:** David Duval, Richard Galinier, Gabriel Mouahid, Eve Toulza, Jean François Allienne, Julien Portela, Christophe Calvayrac, Anne Rognon, Nathalie Arancibia, Guillaume Mitta, André Théron, Benjamin Gourbal

**Affiliations:** 1 CNRS, UMR 5244, Ecologie et Evolution des Interactions (2EI), Perpignan, France; 2 Université de Perpignan Via Domitia, Perpignan, France; 3 Laboratoire de Chimie des Biomolécules et de l’Environnement (LCBE, EA 4215), Perpignan, France; George Washington University School of Medicine and Health Sciences, UNITED STATES

## Abstract

**Background:**

Schistosomiasis is the second-most widespread tropical parasitic disease after malaria. Various research strategies and treatment programs for achieving the objective of eradicating schistosomiasis within a decade have been recommended and supported by the World Health Organization. One of these approaches is based on the control of snail vectors in endemic areas. Previous field studies have shown that competitor or predator introduction can reduce snail numbers, but no systematic investigation has ever been conducted to identify snail microbial pathogens and evaluate their molluscicidal effects.

**Methodology/Principal findings:**

In populations of *Biomphalaria glabrata* snails experiencing high mortalities, white nodules were visible on snail bodies. Infectious agents were isolated from such nodules. Only one type of bacteria, identified as a new species of *Paenibacillus* named *Candidatus* Paenibacillus glabratella, was found, and was shown to be closely related to *P*. *alvei* through 16S and Rpob DNA analysis. Histopathological examination showed extensive bacterial infiltration leading to overall tissue disorganization. Exposure of healthy snails to *Paenibacillus*-infected snails caused massive mortality. Moreover, eggs laid by infected snails were also infected, decreasing hatching but without apparent effects on spawning. Embryonic lethality was correlated with the presence of pathogenic bacteria in eggs.

**Conclusions/Significance:**

This is the first account of a novel *Paenibacillus* strain, *Ca*. Paenibacillus glabratella, as a snail microbial pathogen. Since this strain affects both adult and embryonic stages and causes significant mortality, it may hold promise as a biocontrol agent to limit schistosomiasis transmission in the field.

## Author Summary

The present paper reports the isolation and the characterization of a new microbial pathogen of the freshwater snail, *Biomphalaria glabrata*. Genetic analyses revealed that the species has not been previously described and could be classified into the *Paenibacillus* genus. These bacteria invade most snail tissues and proliferate, causing massive lethality. Moreover, the bacterial infection can be transmitted both vertically and horizontally to other snails, causing their death in 30 days. This discovery is potentially important because *B*. *glabrata*, as an intermediate host, plays an important role in transmitting schistosomiasis, the second-most widespread human parasitic disease. The World Health Organization’s objective of schistosomiasis eradication in a decade encourages the development of multiple approaches for countering the disease, one of which is vector population control. This new bacterial strain clearly could be a potential agent for such a strategy.

## Introduction

Schistosomiasis, the second-most widespread human parasitic disease after malaria, is caused by flatworms of the genus *Schistosoma* (Platyhelminthes, Digenea), currently known to consist of 22 species, three of which *Schistosoma haematobium*, *Schistosoma japonicum*, and *Schistosoma mansoni* are the principal agents of human schistosomiasis. *S*. *mansoni*, the most common causative agent, has a complex life cycle that involves two hosts [1–3]. Adult worms mate in the venous system of a human host, producing eggs that are expelled with the infected person’s feces. If deposited in an aquatic environment, the eggs hatch and each releases a miracidium that can infect *Biomphalaria sp*. a freshwater snail. Inside snail tissues, the miracidium transforms into a primary sporocyst (Sp1) that multiplies asexually to produce secondary sporocysts (Sp2), which then produce cercariae. Cercariae leave the snail and actively infect the vertebrate definitive host.

Because of their medical and epidemiological importance as intermediate hosts for *Schistosoma* parasites, freshwater snails have attracted significant research attention. A number of studies have focused on the immunology of infections transmitted by snails like *Biomphalaria sp*., seeking to identify key genes associated with snail resistance or susceptibility to the parasite. Indeed, several comparative transcriptomic studies have been performed on snails infected by different parasites, such as *Echinostoma* or *Schistosoma* [4–12], or gram-positive or -negative bacteria, like *E*. *coli* [9,13]. Although host snails were shown to develop an immune response against these different potential pathogens, no lethal effects were observed with bacteria even at higher densities of inoculation. To our knowledge, there are no reports of the isolation of bacteria from field samples or under laboratory conditions that are pathogenic towards *Biomphalaria*. However, many efforts were made to find microbial pathogens for use as biocontrol agents to reduce snail populations and thereby control transmission of the parasite. For example, *Bacillus thuringiensis*, a gram-positive, spore-forming bacterium known to secrete many toxins, is widely used against different insect pests [14–16]. Its broad-spectrum action has also been tested against *Schistosoma* vector snails such *Biomphalaria alexandrina*. Although *B*. *thuringiensis israelensis* has no effect [17], *B*. *thuringiensis kurtsaki* has negative effects on snail populations by exerting molluscicidal activity and preventing egg hatching [18]. *Brevibacillus laterosporus* has also been reported to be pathogenic against juveniles of *Biomphalaria glabrata* [19]. A preliminary study suggested the potential pathogenicity of *Bacillus brevis* towards *Biomphalaria pfeifferi* and *Bulinus truncatus* [20], but this has never been tested in the field. *Bacillus pinotti*, isolated from the ovotestis of *Australorbis glabratus* (now re-named *Biomphalaria glabrata)*, has also been examined as a potential biological snail control agent, but results were disappointing [21].

Few studies have examined snails for abnormal symptoms suggestive of infection by microorganisms, such as the presence of nodules. The presence of a mycobacterium (acid-alcohol–resistant microorganism) and a gram-negative bacterium growing as a tumor have been reported in *B*. *glabrata* and *Bulinus jousseaumei*, but neither was associated with signs of pathogenicity [22,23]. Thus, a better knowledge of the potential microbial pathogens of *B*. *glabrata* could contribute to the discovery of new means for preventing and/or controlling schistosomiasis by limiting the vector snail population in the field.

We report the identification of a new snail microbial pathogen named *Ca*. Paenibacillus glabratella. After isolation from infected snails and molecular characterization, we evaluated its pathogenicity against adult snails. The results suggest that this biological agent has potential for vector control of human schistosomiasis.

## Methods

### Ethics statement

For experiments on animals. Our laboratory holds permit #A66040 for experiments on animals from both the French Ministry of Agriculture and Fisheries, and the French Ministry of National Education, Research, and Technology. The housing, breeding, and care of animals utilized here followed the ethical requirements of our country. The experimenter also possesses an official certificate for animal experimentation from both French ministries (Decree #87–848, October 19, 1987). Animal experimentation followed the guidelines of the CNRS (Centre National de la Recherche Scientifique). The protocols used in this study have been approved by the French veterinary agency from the DRAAF Languedoc-Roussillon (Direction Régionale de l’Alimentation, de l’Agriculture et de la Forêt), Montpellier, France (Authorization #007083).

### Biological material

*The B*. *glabrata* strains used in this study were the Venezuelan strain of pigmented *B*. *glabrata* (BgVEN) and the Brazilian strain of unpigmented (BgBRE). The new *Paenibacillus* species was discovered by virtue of its direct association with *B*. *glabrata* snails carrying atypical large, white nodules.

### Bacterial genomic DNA extraction

Bacterial nodules of five BgBRE snails were collected with the aid of an optical microscope and were emulsified in 200 μL of sterile milliQ water. Genomic DNA was extracted from nodules using a PowerLyser UltraClean Microbial DNA Isolation Kit (MO BIO Laboratories), as described by the manufacturer. Briefly, 50 μL of emulsified nodule was resuspended in 300 μL of Microbead solution, which lyses cells through detergent and mechanical actions. After protein precipitation, genomic DNA was selectively bound to a silica-based membrane, washed with ethanol, and eluted with 50 μL of 10 mM Tris buffer at pH 7. DNA concentration was measured using an Epoch micro-volume spectrophotometer system.

### *Paenibacillus* isolation

About three nodules were collected from each of five BgBRE snails with autoclaved dissecting forceps and transferred into 100 μL of sterile milliQ water. After vortexing for 10 minutes at maximum speed, suspended materials were heated for 20 minutes at 75°C to eliminate vegetative microbes. In order to encourage growth of surviving microbes (e.g. spores), inocula were incubated at 25°C or 37°C under aerobic or anaerobic conditions on different media, including LB (Luria Bertani); TSB (trypticase soy boy); brain heart and meat liver infusions; Mueller Hinton supplemented with yeast, phosphate, glucose and pyruvate (MYPGP); and Columbia agars. Only germinated bacteria were present on different media but no bacterial growth was observed under these different conditions. The nodules and the germinated bacteria were picked and investigated by Gram staining and genetic characterization.

### Histopathological examination

Snails presenting symptoms of a bacterial infection were examined histologically. Five infected BgBRE snails were fixed in Halmi’s fixative (90% Heidenhain’s SuSa solution and 10% picric acid-saturated water solution) for 48 hours. Fixed mollusks were then dehydrated successively in two baths of absolute ethanol (24 hours each) and three baths of water-saturated butanol (24 hours each). Dehydrated snails were embedded in paraffin by impregnating for 8 hours. Transverse histological sections (10-μm thick) were cut, mounted on glass slides, then dipped sequentially in toluene (two times for 10 minutes), butanol (10 minutes), and 70% ethanol (5 minutes). After rehydration, slides were treated first with Lugol’s iodine for 30 seconds and then with 5% sodium thiosulfate until bleaching was complete, after which slides were rinsed in distilled water. Rehydrated slides were stained with 0.05% aqueous azocarmine G (Merck, Germany) supplemented with 1% glacial acetic acid for 45 minutes at 60°C, then transferred to a solution of 1% aniline blue in 70% ethanol for 15 minutes. Staining was stopped by addition of 1% acetic acid in 95% ethanol. After treatment with 5% phosphotungstic acid for 30 minutes, slides were rinsed in distilled water and stained with Heidenhain’s azan trichrome for 50 minutes. Preparations were then dehydrated in 95% ethanol for 10 minutes and absolute ethanol for 30 minutes, cleared by immersion in butanol and toluene (50:50 v/v), and mounted with Entellan prior to microscopic examination. Pictures were taken with a Nikon MICROPHOT-FX microscope and a Nikon digital sight DS-Fi1 camera.

### Molecular characterization by polymerase chain reaction

The 16S rRNA gene was amplified by polymerase chain reaction (PCR) using the universal primers 27F (5’-AGAGTTTGATCMTGGCTCAG-3’) and 1492R (5’-ACCTTGTTACGACTT-3’) [24]. To support the identification of a new bacterial species, we also amplified the RNA polymerase beta subunit gene (Rpob) with the primer pair, *Rpob*1698f (5’-AACATCGGTTTGATCAAC-3’) and *Rpob*2041r (5’-CGTTGCATGTTGGTACCCAT-3’) [25–27], and performed PCR using the eukaryotic-specific primer pair, Unif-F-15 (5’-CTCCCAGTAGTCATATGC-3’) and Unif-R-1765 (5’-ACCTTGTTACGACTAC-3’), to amplify the 18S rRNA gene [28]. In each case, thermocycling conditions were 94°C for 8 minutes followed by 35 cycles of 94°C for 30 seconds, 54°C for 30 seconds, 72°C for 2 minutes and a final 5-minute extension step at 72°C. The PCR products were then cloned into the pCR4-TOPO vector according to the manufacturer’s instructions (Invitrogen). The accuracy of cloning steps was confirmed by sequencing of both strands of each clone (GATC Biotech, Germany).

### Phylogenetic analysis

Sequences of ribosomal 16S rRNA and Rpob genes and proteins were retrieved from GenBank (S1 Table). The DNA dataset for the 16S sequences used for phylogenetic analyses contained 26 taxa and 1389 characters. The dataset for the Rpob partial coding sequences contained 22 taxa and 303 characters. The dataset for the Rpob amino-acid sequences contained 25 taxa and 101 characters. Two *Clostridium* species were used as an out-group.

Sequences were analyzed using MEGA version 5.2.2 [29]. Each set of sequences was aligned using Muscle software, and the best substitution model, that is, the one with the lowest Bayesian Information Criterion (BIC), was selected. Phylogenetic trees were constructed using the maximum likelihood (ML) algorithm and tested with the bootstrap method (500 replications). Bayesian analyses were performed using MrBayes 3.2 with four Markov chains for 10^6^ generations. Every 1000^th^ generation was sampled, and the first 25% of trees were discarded; we checked that the standard deviation of the split frequencies fell below 0.01 to ensure convergence of the tree search.

For 16S sequences, the ML tree was computed using the Kimura two parameter model [30] with a proportion of invariant sites (I) along with Gamma-distributed among-site rate heterogeneity (G). For Rpob sequences, the Tamura three parameter substitution model [31] with a gamma-distributed rate heterogeneity was used. The same topology was obtained using the translated amino-acid sequences instead of the DNA sequences. For Bayesian trees, Rpob protein sequences were analyzed using a mixed amino acid model, and 16S nucleotide sequences were analyzed using a general time reversible (GTR) model with gamma-distributed rate variation across sites and a proportion of invariant sites.

### Assay of snail survival after *Paenibacillus* exposure

Sixty uninfected albino *B*. *glabrata* snails originating from Brazil (BgBRE) with shell diameters ranging from 7 to 11 mm were divided in two groups and placed in 3 L of *Ca*. Paenibacillus glabratella-free water. One group of 30 BgBRE snails was exposed to five *Paenibacillus nov*. *sp*.-infected pigmented (BgVEN) snails for 3 months and a second group of 30 BgBRE snails (controls) was exposed to five uninfected BgVEN snails for 3 months. Albino snails were chosen because they facilitated tracking of the spread of bacteria and could be easily separated from infected pigmented snails. Kaplan-Meier survival analyses followed by pairwise log-rank tests were used to compare survival data [32,33]. Infection was determined by direct observation using a binocular stereomicroscope, and mortality was assessed every 2 or 3 days. Dead snails were not removed during the course of the experiment. Snails were fed fresh lettuce twice a week, and half the aquarium water was changed every week. The number of egg masses and the juveniles were measured weekly during the first 28 days before the first observation of mortality.The presence or absence of *Ca*. Paenibacillus glabratella in the rearing water of snails was tested by filtering 1 L of tank water through a 0.22-μm pore-size membrane filter (Millipore). The membrane filter was placed into a 14 mL sterile tube containing 5 mL of sterile water. The tube was vortexed at maximum speed for 3 minutes and then centrifuged for 20 minutes at 4000 × g. The membrane and 4.5 mL of supernatant were removed, and the pellet was suspended in the remaining supernatant by vortexing. The resuspended pellet was 10-fold serially diluted for PCR analysis using universal primers for the 16S rRNA gene and specific primers for the 16S rRNA gene of infectious *Ca*. Paenibacillus glabratella (5’-ATCATCCTCGCATGAGGGAGAT-3’ and 5’-GAGCAGTTCTCTCCTTGTTC-3’). PCR was performed using a hybridization temperature of 43°C and an elongation time of 40 seconds. This pair of primers was also used to verify the absence of *Paenibacillus nov*. *sp*. in healthy snails and confirm its presence in infected eggs and snails.

### Accession numbers

Nucleotide sequence data for *Ca*. Paenibacillus glabratella reported in this paper are available in the GenBank database under the accession numbers KF801672 and KF801673 for the partial 16S rDNA and Rpob genes, respectively.

## Results

### Isolation of a gram-positive pathogenic bacillus from *B*. *glabrata*.

**Fig 1:**
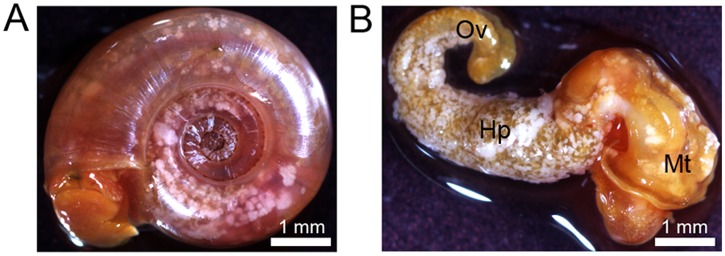
A. Infected *Biomphalaria glabrata* exhibits white nodules. B. Dissected snail presents nodules on mantle (Mt), hepato-pancreas (Hp) and ovotestis (Ov) regions.

**Fig 2:**
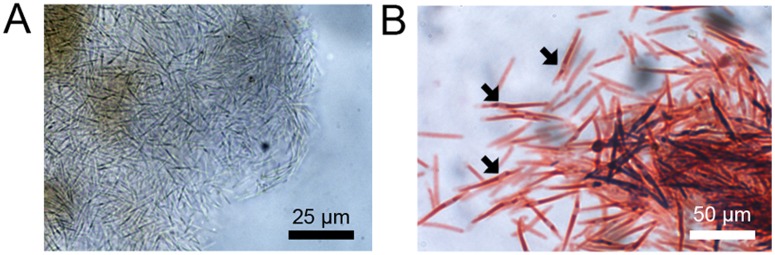
A. An exophytic nodule contains a homogenous population of bacillus- like bacteria. B. These rod-shaped bacteria present a mixed pattern of Gram staining and produce some endospores indicated by the black arrow.

For several years, our laboratory has been a recognized World Health Organization (WHO) collaborating center for the maintenance of the schistosomiasis parasite life cycle and for breeding field-collected snails. During a routine checkup, some snail strains presented with white nodules (Fig. 1A) located on the ovotestis, hepatopancreas, and mantle regions (Fig. 1B). No changes in behavior were observed for these snails, but there was high mortality in breeding tanks. Snails were dissected to isolate and analyze the nature of these white clusters. Under a light microscope, nodules appeared to contain a homogeneous population of microorganisms similar to Ascomyceta or Firmicute phyla (Fig. 2A). Gram staining indicated that all isolates were gram-variable, rod shaped organisms capable of forming subterminal endospores (Fig. 2B).

### Histological characterization of *Paenibacillus* infection

Histological examination of infected snails revealed that pathology was not limited to the external tegument. Indeed, bacterial masses were detected in almost all sections analyzed. Colonies were more or less spherical, depending on their location in the snail tissues. The bacterial colonies were surrounded by collagen-like fibers that formed cyst-shape structures without associated-granuloma or hemocytic nodules.

**Fig 3:**
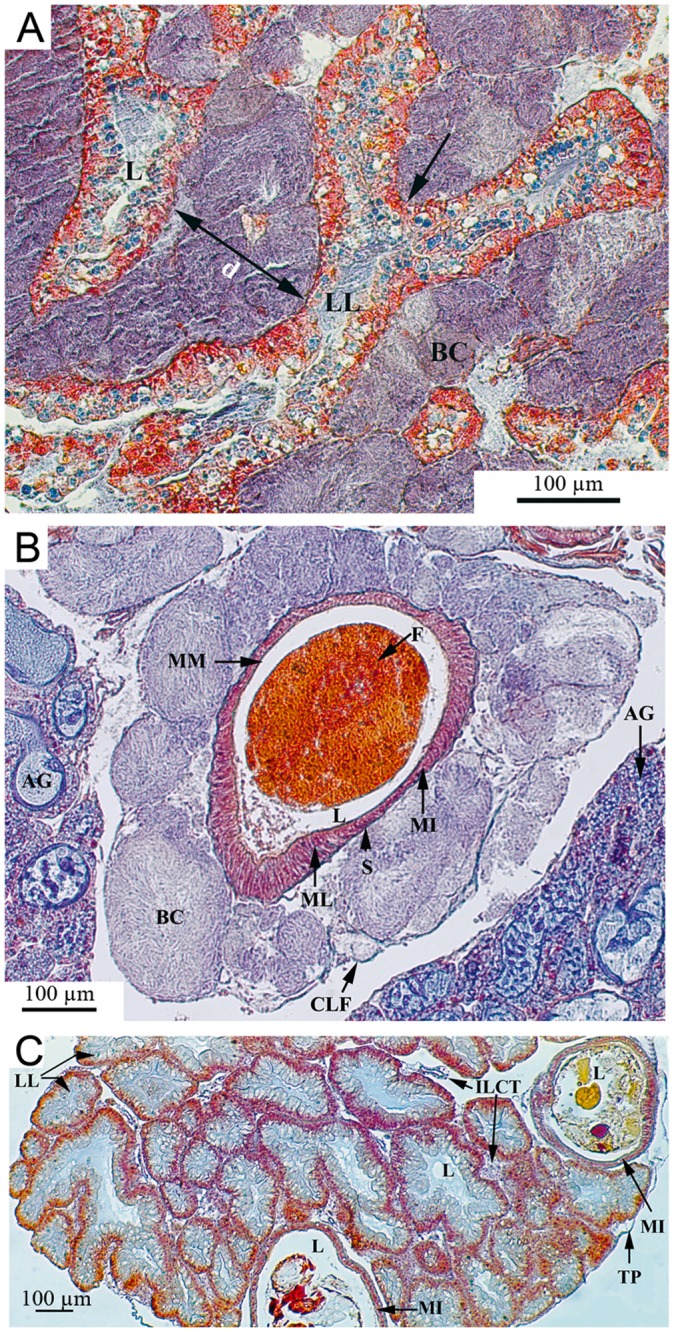
Histological sections of *Biomphalaria glabrata* digestive organs diseased by *Paenibacillus* (A and B) and control (C). (A) The hepatic interlobular space is heavily invaded by the bacterial colonies which separate the hepatic lobules widely (d = 159 μm). The compression exerted (arrows) provoke mechanical damages with degeneration and atrophy of the digestive gland cells. (B) The intestine is densely surrounded by big bacterial colonies causing a slight compression. (C) The control shows normal liver tissue with numerous lobules separated by tight interlobular spaces and normal organization of the midintestine. AG: Albumin gland; BC: Bacterial colony; CLF: Collagen-like fibers; d: Interlobular distance; F: Feces; ILCT: Interlobular connective tissue; L: lumen; LL: Liver lobule; MI: Midintestine; ML: Muscle layer; MM: Mucous membrane; S: Serosa; TP: Tunica propria.

**Fig 4:**
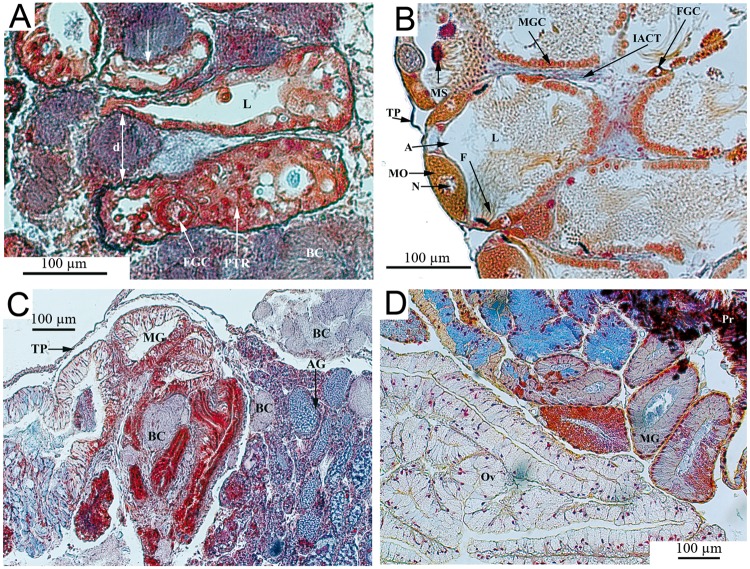
Histological sections of reproductive organs from *Biomphalaria glabrata* infected by *Paenibacillus* (A and C) and control (B and D). (A) The ovotestis interacini space is heavily invaded by the bacterial colonies which exert compression (arrows), separating the acini widely (d = 81 μm). (B) The control shows normal acini organisation in the ovotestis with numerous spermatozoa and some ova. (C) Muciparous and albumin glands are moderately invaded. A: Acinus; AG: Albumin gland; BC: Bacterial colony; d: Interacini distance; F: Flagella; FGC: Female germinal cell; IACT: Interacini connective tissue; L: Lumen; MG: Muciparous gland; MGC: Male germinal cells; MO: Mature ova; N: Nucleus; Ov: Oviduct; Pr: Prostate; PTR: Proliferative tissue response; TP: Tunica propria.

**Fig 5:**
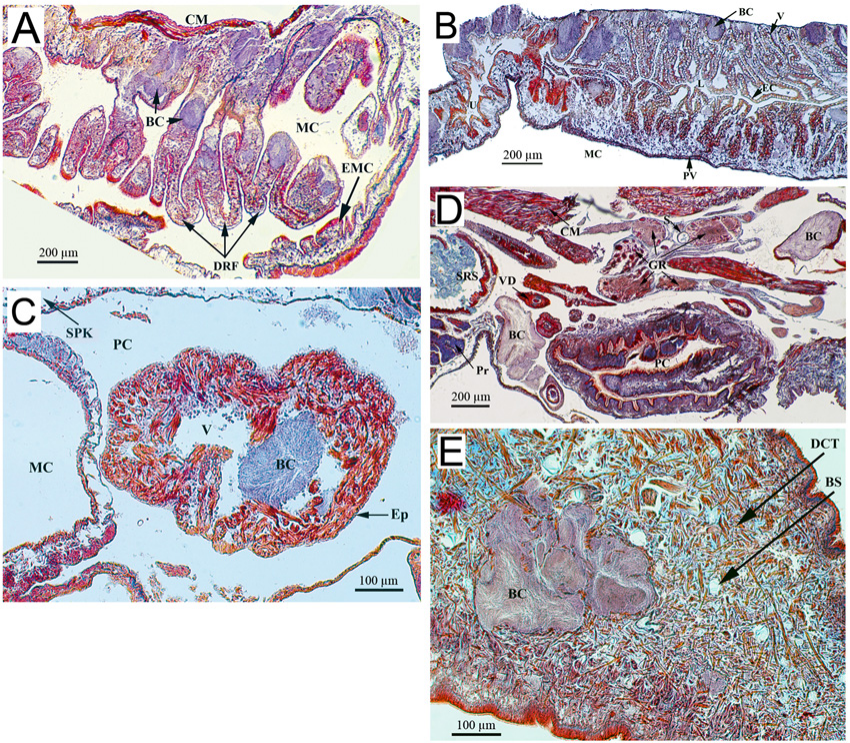
Histological analysis of the bacterial invasion within other organs of *Biomphalaria glabrata*. (A) The dorsal ridge of the mantle cavity with prominent folds invaded by *Paenibacillus* colonies. (B) The kidney tubular portion of *Biomphalaria glabrata* showing *Paenibacillus* colonies exerting compression on the epithelial tissue. The epithelium, constituted by one layer of cells, shows an irregular wavy appearance and a large vacuole in each cell. (C) Presence of *Paenibacillus* colony in the heart cavity. (D and E) Many bacterial colonies are present in the headfoot region with no visible damages on the genitalia organs. BC: Bacterial colony; CM: Columnar muscle; DRF: Dorsal ridge folds; EMC: Epithelium of the mantle cavity; MC: Mantle cavity; EC: Epithelial cells; L: Lumen; PV: Pulmonary vein; U: Ureter; V: Vacuole; Ep: Epicardium; PC: Pericardial cavity; SPK: Saccular portion of the kidney; GR: Ganglion ring; Pr: Prostate; PC: Penial complex; S: Statocyst; SRS: Seminal receptacle sac; VD: Vas deferens; BS: Blood space; DCT: Dense connective tissue.

Organs with the most severe bacteria invasion were the massive hepatopancreas, the gastrointestinal tract (Fig. 3), the hermaphroditic reproductive gland (ovotestis) (Fig. 4), and the dorsal ridge of the mantle cavity (Fig. 5). The interlobular connective tissue of the hepatopancreas (Fig. 3A) and interacini connective tissue of the ovotestis (Fig. 4A) were densely occupied and extremely affected by the mechanical pressure imposed by the bacterial load. Some digestive lobules were severely damaged with visible cellular destruction. The thin layer of connective tissue enveloping the hepatopancreas was ruptured. The walls of reproductive acini showed increased thickness with physiological suppression of gamete production (gametogenesis); no spermatozoa were detected in the acinar lumen (Fig. 4A). The dorsal ridge of the mantle cavity (respiratory organ) showed many bacterial colonies in the epithelial tissue of different folds (Fig. 5). A large number of bacterial colonies of different sizes surrounded the intestinal tract and stomach (Fig. 3B). A few bacterial colonies were observed inside sex accessory organs, such as muciparous and albumin glands (Fig. 4C). Saccular and tubular portions of the kidney were moderately infected, with bacterial colonies mainly concentrating in the dorsal area of the kidney and developing towards the kidney lumen (Fig. 5A). Very few bacterial colonies were located on the ventral side of the kidney overlooking the mantle cavity (Fig. 5B). Hematopoietic tissue located between the saccular part of the kidney and the pericardium was also infected. No bacterial colonies were observed in the pericardial cavity; however, infection was detected in cardiac chambers, including a ventricle containing one colony that appeared to occupy a large volume (Fig. 5C). The headfoot sinus was moderately infected by bacterial colonies without any visible damage to male or female genitalia that end in this area (Fig. 5D). Irregularly shaped bacterial colonies were observed in the foot region (Fig. 5E). No necrosis of snail tissues was observed.

### Molecular identification of *Paenibacillus* and phylogenetic analysis

In order to identify the microbial pathogenic agent, we performed a PCR analysis of genomic DNA extracted from the white nodules using universal bacterial primers (27f and 1492r) and eukaryotic-specific primers (Unif-F-15 and Unif-R-1765). No amplification was obtained with eukaryotic-specific primers, whereas one ~1400-bp fragment with a single sequence was amplified using primers for the 16S rRNA gene. This sequence was compared against the NCBI database of 16S ribosomal RNA sequences using a BLASTN search. The best hits were for the 16S rRNA genes of *Paenibacillus alvei* (accession number NR_113577.1) and *Paenibacillus apiarius* (accession number NR_040890.1) with 95% and 94% identity, respectively. In order to confirm this identification, we partially amplified the RNA polymerase beta subunit gene (Rpob) using previously reported primers for the Rpob gene of different *Paenibacillus* species [26,27], and compared the predicted amino acid sequences against the NCBI protein database using a BLASTP search. The most closely related protein sequences were from *Paenibacillus thiaminolyticus* (accession number AY728285.1), with 94% similarity and 87% identity (E-value = 6.10^–62^), and *P*. *alvei* (accession number WP 005544566.1), with 96% similarity and 94% identity (E-value = 9.10^–59^).

**Fig 6:**
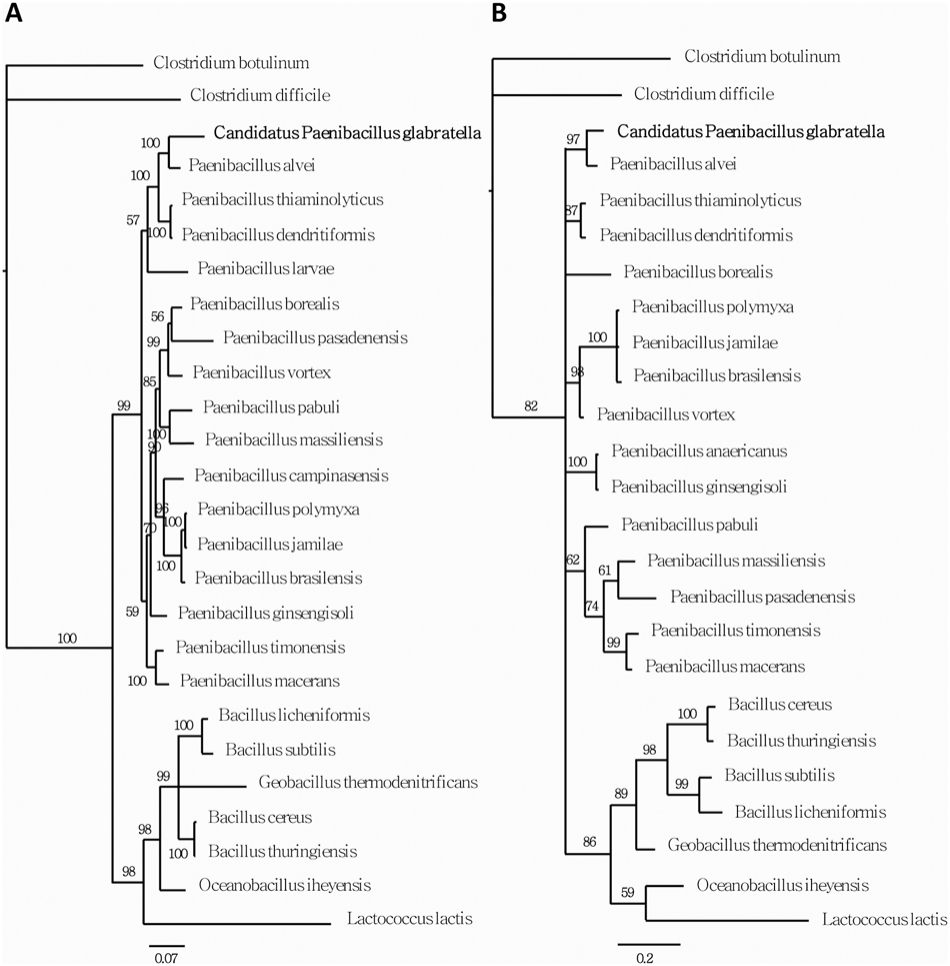
Molecular phylogenies using Bayesian analysis of (A) 16S nucleotide sequences and (B) Rpob aminoacid sequences. Numbers above nodes represent posterior probabilities recovered by the Bayesian analysis.

The family relationships of the infectious agent described in this work were clarified by constructing phylogenetic trees. Similar topologies were obtained using different methods and datasets. The most terminal nodes were highly supported, whereas the relationships among the different families were less consistent, with lower concordance across the two 16S and Rpob datasets (Fig. 6). Nevertheless, these analyses established a new pathogenic bacteria clade within the *Paenibacillus* genus that is most closely related to *P*. *alvei*. With an identity rate of 94% between 16S rDNA sequences, the results of this phylogenetic analysis are indicative of a new species that we call *Candidatus* Paenibacillus glabratella. As recommended for noncultivable bacteria, the provisionnal *Candidatus* status was retained for this newly described *Paenibacillus nov*.*sp*. [34].

### Assay of bacterial pathogenicity against *B*. *glabrata*

As a first step toward testing the virulence of this new bacterial species, we attempted to develop an appropriate growth media. Various types of liquid growth media, including LB, TSB, MYPGP, brain heart or meat infusions, were tested. Before culturing, samples were heat-shock treated to eliminate most vegetative bacteria. No bacterial growth was obtained under aerobic or anaerobic conditions at 27°C or 37°C, even if media were supplemented with 10% filtered, crushed-snail extract. However, bacteria could be maintained on these different agar media with atypical colony formation, suggesting a swarming behavior characteristic of some gram-positive bacteria (S1 Fig.). Bacterial clumps on agar plates were characterized by PCR and found to be identical to those from nodules.

**Fig 7:**
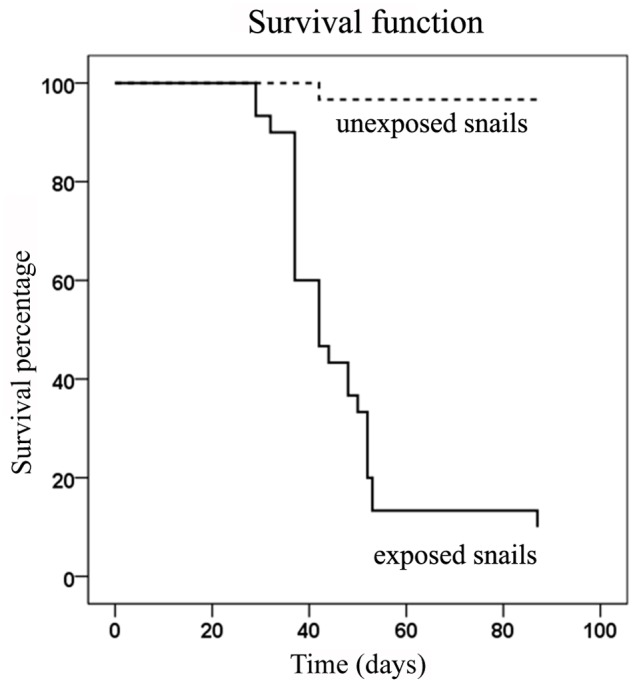
Survival time of *B*. *glabrata* (BgBRE) exposed to either uninfected (a) or bacteria-infected snails (BgVEN). A Kaplan-Meier analysis was performed to analyze survival data.

**Fig 8:**
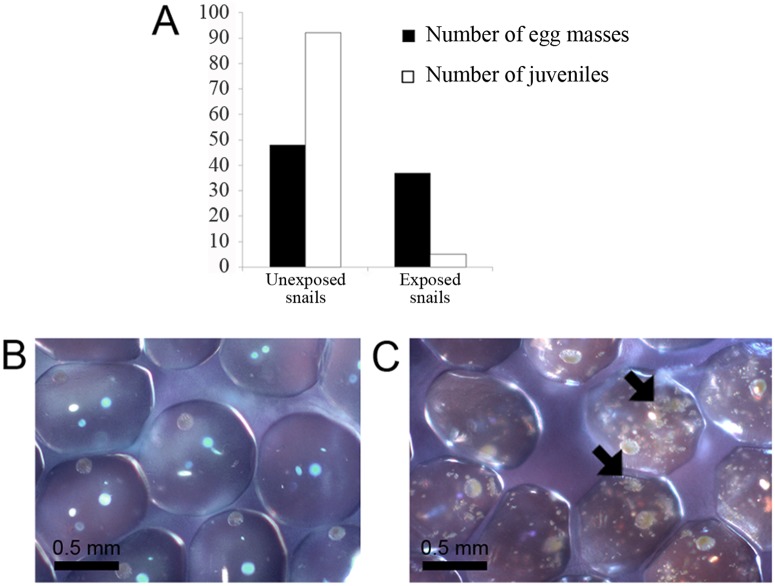
A. Effect of *Candidatus* Paenibacillus glabratella exposure on egg masses and juvenile snails production. B and C. Photomicrographs of egg masses from unexposed and exposed snails, respectively. The arrow indicates the presence of *Ca*. Paenibacillus glabratella inside each egg from exposed snail population.

Because this novel strain exhibited no growth under our culture conditions, we decided to follow its pathogenicity by exposing healthy BgBRE albino snails to infected pigmented (BgVEN) snails. In our experimental conditions, infected snails had many modules, and exposed snails developed multiple detectable nodules after about 1 month. Fifteen days later, the number of dead snails increased sharply, reducing the original population approximately in half. By 30 days (60 days after the initial exposure), about 90% of the population had been eliminated. A Kaplan-Meier analysis confirmed the statistical significance of this result (p<0.0001) (Fig. 7). All dead snails exposed to infected snails presented with visible white nodules as clinical signs. Interestingly, before the period of high mortality (i.e., until 30 days post-exposure), a significantly lower number of juveniles was observed in containers of exposed snails, despite the fact that the number of egg masses was almost identical to that in the control group (Fig. 8A). Indeed, there were 20-times more juveniles in the control container than in the container containing bacteria. This lack of juveniles was correlated with the presence of *Ca*. Paenibacillus glabratella inside the snail eggs, as confirmed by PCR (Fig. 8B and C).

## Discussion

In May 2012, WHO called for the eradication of schistosomiasis and encouraged an integrated approach including chemotherapeutic control, hygiene and health education, and snail control [35]. To date, mass application of praziquantel has proven to be the most effective approach for reducing schistosomiasis prevalence and associated morbidity [36,37]. However, there is growing concern about the emergence of parasite resistance to praziquantel, and unfortunately, no vaccines against schistosomes are currently available [38,39]. A number of molluscicides have been developed in an attempt to decrease parasitic disease transmission. Chemical compounds, such as niclosamide and sodium pentachlorophenate, have been field tested and proven effective [40–43], although concerns about the emergence of a resistant snail population remain [44]. However, even if effective in decreasing the host snail population, these synthetic molluscicides can be toxic to other living organisms [45–47] and are transferred up the food web [48,49]. Consequently, increasing efforts have focused on identifying and characterizing plant-derived molluscicides as safer alternatives. Compounds belonging to saponin and terpenoid classes are highly effective against adult snails, but in most cases, the effects of these compounds on egg hatching are unknown; where they have been tested on egg masses, they have demonstrated lethal effects with higher concentrations than used against adult snails [50–59]. Other plant-derived molecules have effects on snail embryogenesis, but appear to be toxic to non-target animals [60,61]. Another “ecological” strategy for eliminating vector snails and preventing schistosomiasis transmission is the introduction of a competitor or predator into endemic sites. The *Biomphalaria* population is strongly suppressed in Guadeloupe, Puerto Rico and some parts of Brazil after invasion of freshwater snails like *Melanoides tuberculata* or *Marisa cornuarietis* [62–68]. The presence of predators, such as fish, insects or crustacean species, has also proved effective in limiting snail populations, but their effects are generally tested in the laboratory; thus, predator-prey population dynamics under environmental conditions are not known [69–73]. Another snail control strategy that has been considered is the reduction of reproductive capacity. The nematode *Angiostrongylus cantonensis* and trematode parasites belonging to the genus *Plagiorchis* or *Echinostoma* are known to affect growth as well as fecundity and fertility of *B*. *glabrata*, and have thus been proposed as biological control agents [74–76]. However, their use is not recommended since they may also cause severe human or animal infections. Efforts to control other parasitic diseases, such as malaria, and arthropod pests and vectors have focused on micropathogen identification [77–84]. Although WHO proposed studies on snail host microbial pathogens as part of its research proposal guidelines in 1984 [85], few such studies have been conducted [17–19].

In the current study, we report the isolation of a new pathogenic bacterial species for *Biomphalaria*, the snail intermediate host of *S*. *mansoni*. 16S rDNA and Rpob genes sequence analyses positioned this bacterium within the genus *Paenibacillus*. The highest identity rates for 16S rDNA and Rpob genes were only 95% and 94%, respectively, compared with *Paenibacillus alvei* genes. Since these identity levels are well below the threshold for genomic definition of a bacterial species based on the 16S rDNA gene (i.e., 97%), which is commonly used for molecular systematics [86,87], we propose to call this new pathogenic bacterium, *Ca*. Paenibacillus glabratella.

Paenibacillaceae family members are widely distributed in the environment, including in soil, air, the rhizosphere, food products, and aquatic environments [88–90]. Up to date, the exact origin of this new microbial pathogen named *candidatus* Paenibacillus glabratella is unknown. Screening of healthy snails for bacterial agents using a molecular approach excluded the possibility of a snail microbiota origin for the isolated bacterium. Moreover, all bacterial communities characterized and cultivated from *B*. *glabrata* were gram-negatives [91], and everything that came in contact with the mollusk (e.g., water, food) tested negative for *Paenibacillus*, strongly suggesting contamination of animals collected in the field and horizontal transmission between laboratory snails. Indeed as WHO collaborating center we recovered in the field and reared in our laboratory a great number of snail strains from different localities (mainly South America and Africa). One of these snail isolates could have been the point of entry of the disease in our laboratory, but we were not able to identify the strain of origin. In our experimental conditions, the duration to the first signs of mortality is longer than the time period reported in other studies, in which lethal effects appeared after 1 or 2 weeks [18,19]. However, instead of using massive exposure, as was done in these previous studies, we enabled bacterial infection by dissemination from snail to snail using a few initially infected individuals. This horizontal transmission was surprisingly effective, resulting in the death of almost all exposed snails.

Among bacteria belonging to the family Paenibacillaceae, only *P*. *larvae* and *P*. *popilliae* are described as invertebrate pathogens [92–94]. The first is a spore-forming bacterium that is a widespread larval pathogen of the honey bee, identified as the ethological agent for American foulbrood [95,96]. After being ingested in the form of a spore by honeybee larvae, this bacterium grows in the midgut of the insect. Then, the vegetative form secretes proteases that facilitate tissues invasion and contribute to liquefaction of the host [97,98]. The most marked histopathological feature of *Paenibacillus* interference with *B*. *glabrata* is its development in a large number of tissues, mainly in the hepatopancreas, that compromises the ability of the snail to nourish itself. The presence of *Paenibacillus* in the circulatory system, mainly in the heart, indicates that these bacteria may follow the path of the hemolymph to reach different organs involved in diverse functions, including digestion, respiration, excretion, and reproduction. In keeping with this, *Paenibacillus* was observed in the ovotestis, leading to the suppression of gametogenesis and partial destruction of ovotestis acini. So, the snail reproductive capacity could be affected by compromising the number of eggs laid in the advanced stage of the infection. A castration-like phenomenon similar to that observed in heavily infected snails has also been reported during parasitic infestation by *S*. *mansoni* [99,100], *Plagiorchis elegans* [74], and the nematode *Angiostrongylus cantonensis* [76]. This late infertility appears to be related to competition for energy resources between the host and pathogen [76,100]. *Paenibacillus* was also observed in the secondary reproductive organs, namely muciparous and albumin glands, which could be a sign of vertically transmitted infection from parent to offspring. Indeed, this pathogenic agent was also found in snail eggs, affecting their development and hatching. Collectively, these histological observations suggest that the main pathogenic effect of *Ca*. Paenibacillus glabratella was strong compression of tissues, which caused significant damage to soft tissue organs like the liver and ovotestis. However, we do not exclude the possibility of tissue lysis owing to bacterial excreta, as it has been observed for *Brevibacillus laterosporus* towards nematodes [101], or gametogenesis inhibitory bacterial factors, as has been reported for the digenean trematode *Zoogonus lasius* on the snail, *Nassarius obsoletus* snail [102].

In conclusion, a newly recognized pathogenic bacterium that is closely related to members of the genus *Paenibacillus* was isolated from abnormal nodules of the snail *B*. *glabrata* and characterized. Although this microbial pathogen could only be cultivated *in vivo* (*in vitro* cultivation conditions have not yet been established), we demonstrated that it is infective through aquatic dispersal and contact between snails. Upon infection, this bacterium is highly lethal for adult snails and severely reduces the number of offspring. Our current report describes the pathogenic effects of this bacterium on the neotropical snail *B*. *glabrata*, but the African snail *B*. *pfeifferi* and the genus *Bulinus*, other *Schistosoma* vectors, can also be affected (personal data). Among important future studies are tests of the spectrum of the molluscicidal properties of this bacterium against all freshwater snails that can serve as vector for schistosomiasis. However the high pathogenicity of this bacterium could be a limit for using it as biological control agent. Indeed its safety for non target species has also to be tested to avoid deleterious impact on endemic species. Finally, it would also be interesting to study the pathogenicity of other species that are *closely related phylogenetically to Ca*. Paenibacillus glabratella, like *P*. *alvei* [103]. Clearly, data obtained in the laboratory must be confirmed under field conditions including non-target species before proposing this pathogen as a biocontrol agent against schistosomiasis. Nevertheless, this study highlights the value of systematic surveys of snail pathology in providing potential tools to support the announced WHO goal of schistosomiasis eradication by 2025 [35,104].

## Supporting Information

S1 Table Accession numbers of the sequences used for phylogenetic analyses. Sequence accession numbers of the species used for the phylogenetic analysis. The table provides the GenBank accession number of nucleotide sequences from 16S rDNA and RpoB genes and Rpob protein sequence.
(DOCX)

S1 Fig *Candidatus* Paenibacillus glabratella on LB agar plate under anaerobic condition. Individual cells and clump of bacteria interconnected by long strings forming some hyphae like structure were observed. The arrow shows an interconnection between two bacteria clumps.
(TIF)
