# Correction: A Novel Bacterial Pathogen of *Biomphalaria glabrata*: A Potential Weapon for Schistosomiasis Control?

**DOI:** 10.1371/journal.pntd.0003815

**Published:** 2015-06-15

**Authors:** David Duval, Richard Galinier, Gabriel Mouahid, Eve Toulza, Jean François Allienne, Julien Portela, Christophe Calvayrac, Anne Rognon, Nathalie Arancibia, Guillaume Mitta, André Théron, Benjamin Gourbal

There is an error in the penultimate sentence of the “Assay of snail survival after *Paenibacillus* exposure” subsection of the Methods section. The second primer in the parentheses should be GAGCAGTTTCTCTCCTTGTTC.
