# Supplementary material for: A Novel Bacterial Pathogen of Biomphalaria glabrata: A Potential Weapon for Schistosomiasis Control?
Source: PLoS Negl Trop Dis. 2015 Feb 26;9(2):e0003489. doi: 10.1371/journal.pntd.0003489 (PMC4342248; doi:10.1371/journal.pntd.0003489)
Supplement: S1 Table — Sequence accession numbers of the species used for the phylogenetic analysis. The table provides the GenBank accession number of nucleotide sequences from 16S rDNA and RpoB genes and Rpob protein sequence. (DOCX) [file pntd.0003489.s001.docx]

**Table S1: Accession numbers of the sequences used for phylogenetic analyses.**

| Species | 16S | Rpob (nt) | Rpob (aa) |
| --- | --- | --- | --- |
| Bacillus subtilis | DQ195067.1 | CP002183.1 | YP_003864477.1 |
| Bacillus cereus | NR_074540.1 | FJ188319.1 | FJ188319.1 |
| Bacillus licheniformis | DQ993676.1 | AF172323.1 | AAD48492.1 |
| Bacillus thuringiensis | CP000485.1 | CP000485.1 | YP_893022.1 |
| Clostridium botulinum | NR_036786.1 | Y16466.1 | CAC10529.1 |
| Clostridium difficile | NR_074454.1 | AM180355.1 | YP_001086530.1 |
| Geobacillus thermodenitrificans | AB546234.1 | EU484374.1 | ACB12773.1 |
| *Candidatus* Paenibacillus glabratella | KF801672 | KF801673 | KF801673 |
| Lactococcuslactis | FJ348447.1 | AF531271.1 | AAM94427.1 |
| Oceanobacillus iheyensis | NR_075027.1 | BA000028.3 | NP_691033.1 |
| Paenibacillus alvei | NR_042091.1 | NA | WP_005544566.1 |
| Paenibacillus macerans | NR_040886.1 | AY493863.1 | AY493863.1 |
| Paenibacillus timonensis | NR_042844.1 | AY728289.1 | AY728289.1 |
| Paenibacillus anaericanus | FN673683.2 | FN673683.2 | FN673683.2 |
| Paenibacillus borealis | AJ011322.1 | AY493866.1 | AY493866.1 |
| Paenibacillus brasilensis | JQ860094.1 | HE972657.1 | HE972657.1 |
| Paenibacillus dendritiformis | NR_042861.1 | AY728286.1 | AY728286.1 |
| Paenibacillus ginsengisoli | FN673695.1 | FN673684.3 | FN673684.3 |
| Paenibacillus jamilae | HQ844448.1 | HE972655.1 | HE972655.1 |
| Paenibacillus massiliensis | NR_029098.1 | AY728294.1 | AY728294.1 |
| Paenibacillus pabuli | KF055007.1 | AY728291.1 | AY728291.1 |
| Paenibacillus pasadenensis | AB681404.1 | HQ596205.1 | HQ596205.1 |
| Paenibacillus polymyxa | AY359636.1 | HE972654.1 | HE972654.1 |
| Paenibacillus thiaminolyticus | NR_040887.1 | AY728285.1 | AY728285.1 |
| Paenibacillus vortex | HQ005270.1 | NA | WP_006212933.1 |
