# Supplementary figures and images for: A Novel Bacterial Pathogen of Biomphalaria glabrata: A Potential Weapon for Schistosomiasis Control?
Source: PLoS Negl Trop Dis. 2015 Feb 26;9(2):e0003489. doi: 10.1371/journal.pntd.0003489 (PMC4342248; doi:10.1371/journal.pntd.0003489)

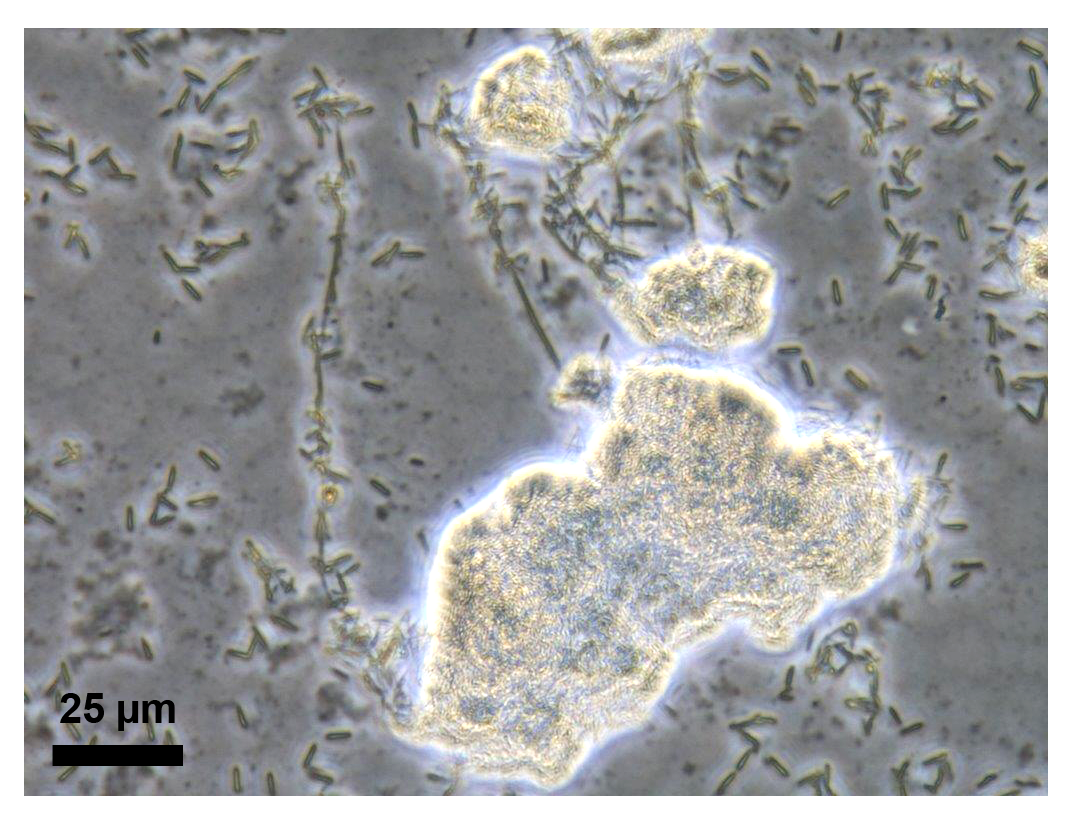

Supplement: S1 Fig — Individual cells and clump of bacteria interconnected by long strings forming some hyphae like structure were observed. The arrow shows an interconnection between two bacteria clumps. (TIF) [file pntd.0003489.s002.tif]
